# Animal-Assisted Intervention and Health Care Workers’ Psychological Health: A Systematic Review of the Literature

**DOI:** 10.3390/ani12030383

**Published:** 2022-02-04

**Authors:** Daniela Acquadro Maran, Ilaria Capitanelli, Claudio Giovanni Cortese, Olayinka Stephen Ilesanmi, Maria Michela Gianino, Francesco Chirico

**Affiliations:** 1 Department of Psychology, Università di Torino, 10124 Torino, Italy; daniela.acquadro@unito.it; 2 Prevention Service in the Workplace (SPRESAL), Local Health Unit Roma 4, 00053 Civitavecchia, Italy; ilaria.capitanelli@yahoo.it; 3 Department of Community Medicine, College of Medicine, University College Hospital, Ibadan PMB 5116, Nigeria; ileolasteve@yahoo.co.uk; 4 Department of Public Health and Paediatrics, Università di Torino, 10126 Torino, Italy; mariola.gianino@unito.it; 5 Post-Graduate School of Occupational Health, Università Cattolica del Sacro Cuore, 00168 Roma, Italy; medlavchirico@gmail.com

**Keywords:** health care workers, animal-assisted intervention, burnout, psychological well-being

## Abstract

**Simple Summary:**

In health care, animal-assisted intervention has been used primarily to enhance the positive effects of therapy. For example, it has been used with patients suffering from autism spectrum symptoms, medical difficulties, behavioral problems, and emotional disorders. More recently, this type of intervention has been increasingly used in the workplace to mitigate the effects of stress in employees (including healthcare workers). The aim of this systematic review was to analyze the potential benefits of animal-assisted intervention in healthcare workers.

**Abstract:**

Healthcare settings have recently increased the use of companion animals in the workplace to provide emotional support to people with disabilities, but there is limited empirical research on the effects of these programs on healthcare workers. However, it is reasonable to speculate that Animal-Assisted Interventions (AAIs) may have positive effects on health care workers (HCWs) by buffering the negative effects of work-related stress and other occupational psychosocial risk factors. The aim of this review was to examine the beneficial effects of AAIs on the psychological well-being of HCWs. A systematic review was conducted in December 2021 to gain insight into the positive effects of pets on HCWs in the workplace. Searches were conducted in the following databases: Scopus, PubMed/Medline, Web of Science, and Google Scholar, including studies between 2001 and December 2021, and 12 articles were included in the review. The results indicate that implementing the AAI program in a busy clinic is feasible and that the program is accepted by medical professionals because of the immense psychological benefits it provides. However, the healthcare professionals disliked the experimental design that forced them to leave their workplaces at a certain time.

## 1. Introduction

Psychosocial risks (e.g., work-related stress, emotional demands, and workplace violence) pose a significant threat to workers’ physical and mental well-being and are a tremendous burden on organizations and economies [1,2]. Work-related stress (WRS) is the negative reaction that people may have when there is a discrepancy between job demands that require effort in the form of cognitive and emotional responses and the worker’s perception of being able to cope with them [3]. Short-term exposure to WRS can improve individual abilities by increasing attention and concentration, but prolonged exposure to intense pressure can overwhelm workers’ coping resources and lead to physiological, psychological, and social consequences. At the individual level, WRS can cause physical disorders, such as hypertension, digestive and sleep disorders, as well as low back pain and musculoskeletal symptoms [4,5]. WRS, emotional demands, and workplace violence have even been linked to mental health disorders, such as depression, anxiety, irritability, confusion, and chronic stress [6,7], sometimes leading to burnout syndrome (BOS) and post-traumatic stress disorder (PTSD). In 2019, BOS was officially recognized in the ICD-11 as “an occupational phenomenon characterized by feelings of energy depletion or emotional exhaustion, increasing mental detachment from work, or feelings of negativism or cynicism about work and, consequently, decreased occupational efficiency” [8,9,10]. Post-traumatic stress disorder at work (PTSD) has been associated with negative working conditions, particularly frequent occupational or emotional trauma, negative interpersonal relationships, and workplace violence by supervisors, colleagues, or third parties [11,12]. These psychosocial risk factors require employers to develop preventive measures, as rehabilitation is complex and medical and cognitive behavioral therapies are not always effective [13,14]. Depression, BOS, and PTSD have been described primarily in caring professions, such as health care workers (HCWs) [15,16,17,18,19]; this is a profession where emotional demands and interpersonal relationships are central parts of the job [20], placing high and challenging demands, including exposure to death and occupational trauma [21]. Studies conducted prior to the COVID-19 pandemic reported that BOS affected up to 20% of healthcare workers [8]. The COVID-19 pandemic has added an additional burden, as the nature of healthcare workers’ activities and contact with the public pose an increased risk of infection to COVID-19 [22,23]. As a result, acute stress disorder and PTSD may occur because of the fear of infection and transmitting the infection to loved ones, high workload, and reduced rest periods, resulting in a high burden of emotional distress [24,25]. As a result, HCWs are experiencing high levels of mental distress, with high rates of anxiety, depression, fear, BOS, and sleep disorders [24,25,26]. For this reason, it was found that the COVID-19 pandemic may be considered as a mass traumatic event with negative consequences on the mental well-being of HCWs and caregivers [27], and solutions for preventing, in the future, increased rates of suicides are needed.

At the organizational level, high levels of WRS disruption in health care workers can lead to increased presenteeism, sick leave, high turnover, frequent interpersonal conflict, lost productivity, low performance, and ultimately high costs to health care institutions [28,29,30]. Conversely, a psychologically safe and healthy workplace actively promotes the emotional well-being of employees, who exhibit greater job satisfaction, improved team learning behaviors, and higher performance, morale, and engagement. Therefore, the implementation of organizational interventions aimed at protecting and promoting mental health in all work environments should be prioritized by healthcare organizations [31,32]. Furthermore, stress management interventions may yield long-term benefits to the quality of care provided [33]. 

To mitigate the negative effects of WRS in workers, Cooper and Cartwright [34] described a three-tiered approach: primary prevention (aiming to eliminate potential sources of stress), secondary prevention (aiming to manage stress after it has occurred), and tertiary prevention (aiming to treat illnesses that result from high levels of WRS). As described by Foreman et al. [35], to promote primary prevention, several companies have implemented policies that allow employees to bring their pets to work (see also [36,37]) to reduce WRS. Previous research has shown that pet owners experience a variety of physical, psychological, and social benefits [38,39,40]. Beetz et al. [41] reported that human–pet interaction positively affects social attention and behavior, interpersonal interactions, and mood. According to Wells and Perrine [39], pets could serve as a “stress buster”: petting or looking at a pet can help to lower blood pressure and heart rate (in the short term). In the long term, this activity may help improve cardiovascular fitness (e.g., [42,43]). In addition, attachment to a pet can increase self-efficacy and self-esteem, and encourage owners to feel positive emotions, which in turn positively affect their coping strategies for managing stress [44,45]. Generally, the presence of pets helps people to cope with stressful tasks by reducing their anxiety and hyperactivity. For example, Polheber et al.’s [46] study involving 48 undergraduate students found that the presence of a dog resulted in lower psychological distress during stressful tasks [38,46] and lower levels of stress-related biological parameters, such as cortisol, heart rate, and blood pressure [35,47,48,49]. As Rehn and Keeling [50] elaborated, pet owners feel less stressed by corporate pet policies than when they have to leave their pets at home for extended periods of time. This is because the source of stress is not only the inability to be with the pet, but also the thought that the pet might suffer from being separated from the owner; this spillover effect between ‘work and family’ and ‘family and work’ has been described by Gershon et al. [51] and may be a source of distress for pet owners. Companies that allow pets to be brought to work are thus not only implementing a primary prevention strategy, but also a secondary prevention strategy, namely the ability to manage stress after it has already set in. Human–pet interaction in a therapy setting is referred to as Animal-Assisted Intervention (AAI) and consists of activities in which “animals are intentionally used in health, education, and human services (e.g., social work) for the purpose of therapeutic benefits to humans (…) AAIs include human–animal teams in human services such as animal-assisted therapy (AAT), animal-assisted education (AAE), or animal-assisted activity (AAA)” ([52], p. 5). AAI has several positive effects on individuals, such as an increase in oxytocin and a decrease in depression symptoms; pets in the workplace help employees to cope better with stress, even when they do not own them [46,53,54,55]. In addition, pets are used in the treatment of stress-related illnesses (tertiary prevention) to increase the benefits of therapy and reduce occupational common trauma. For example, the study by Foreman et al. [35] demonstrated the potential benefits of dogs in the workplace, providing social support to employees by reducing occupational stress levels and improving work performance and engagement. 

In healthcare, companion animals have recently been increasingly used in the workplace to provide emotional support for people living with disabilities [49], but there is little empirical research on the effects of these programs on HCWs. To better understand the potential role of pets in healthcare settings, Gaudet et al. [56] conducted a meta-analysis on the potential benefits of pet presence in alleviating stress among HCWs, patients, and parents/caregivers. Their findings were inconsistent due to some incongruence between qualitative and quantitative studies, so no clear conclusion could be drawn about the benefits of keeping pets in this particular occupational setting. However, it is reasonable to assume that AAIs might have positive effects in HCWs by buffering the negative effects of work-related stress and other psychosocial risk factors.

The aim of this review was, therefore, to examine the psychological impact of AAIs on the psychological well-being of HCWs. To this end, we considered all the occupational interventions used for primary, secondary, and tertiary prevention in healthcare settings.

## 2. Materials and Methods

### 2.1. Search Strategy

Searches were conducted on the following databases: Scopus, PubMed/Medline, Web of Science, and Google Scholar, including studies between 2001 and December 2021. The search strategy combined keywords based on health care workers or profession (population), AAI (intervention), non-healthcare worker (comparison), and anxiety, depression, work-related stress, BOS, PTSD, or other mental health disorder (outcome) in accordance with the elements Population, Intervention, Comparison, and Outcome as follows: (“health care workers” OR “health care providers”) AND (“pets” OR “companion animals” OR “dogs”) AND (“animal-assisted therapy” OR “animal-assisted interventions” OR “animal-assisted activity”), AND (“work-related stress” OR “workplace health” OR “employee well-being” OR “burnout”).

A systematic review was conducted in December 2021 to provide insight into the positive effects of pets on HCWs in workplace settings. The study protocol for this review was registered at PROSPERO on 13 December 2021 under the following registration number: CRD 298027.

### 2.2. Study Selection 

This study was conducted in accordance with the Preferred Reporting Items for Systematic review and Meta-Analyses (PRISMA) [57] guidelines for therapeutic purposes (i.e., AAI in health care and its effects on workers’ psychological health, including work-related stress, distress, burnout, mood change, or work satisfaction). We excluded editorials, reviews, commentaries, guidelines, and articles from the press. We also excluded studies conducted on non-occupational cohorts (e.g., studies on the clinical benefits of AAI in individuals with psychiatric disorders) or conducted among other groups of workers, such as the military, veterans, or social workers. We excluded studies that examined the presence of animals in the workplace without therapeutic purposes (workers who brought their companion animals to work) and studies that examined the psychological benefits of pet ownership or pet attachment, or how pets help people to cope with everyday stressors. Studies that only examined HCWs’ knowledge or attitudes towards AAI were also excluded. Only original studies (both quantitative and qualitative research) in English were included. To retrieve a wide range of literature, the reference lists of the included literature were reviewed to identify further suitable studies.

**Figure 1:**
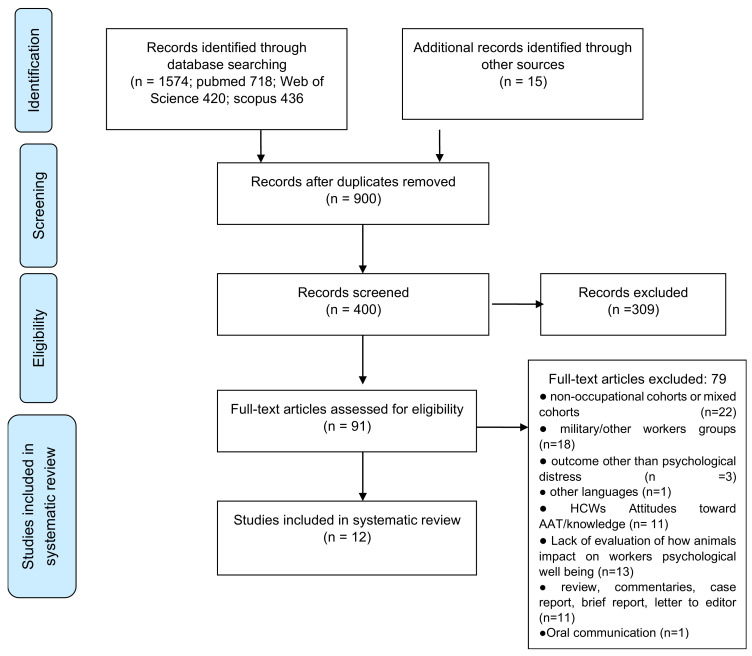
Article selection algorithm (PRISMA).

After independently reviewing all titles/abstracts to identify potentially relevant articles, two authors (I.C. and F.C.) selected studies based on a full-text review by using the above inclusion/exclusion criteria. Disagreements were resolved by a third author (D.A.M.) who acted as the final reviewer. The information extracted included: (i) source (first author and year of publication); (ii) general study details (study design); (iii) setting (country/region considered, study population vs. comparison group, and type of employment); (iv) exposure details (schedule and pattern of AAI); and (v) main study findings. Figure 1 summarizes the article selection strategy.

#### Data Analysis and Study Quality Assessment

After selecting studies, we extracted the necessary data into a form designed to summarize the included studies. The authors conducted the process of data extraction independently. In this review, the findings were narratively analyzed for synthesizing two different forms of evidence, namely qualitative and quantitative studies [58].

The results of the studies were qualitatively evaluated using the checklist proposed by Tufanaru et al. [59]. The checklist provides a quality assessment of the effectiveness of the studies for both qualitative and quantitative studies.

The final results of the quality assessment resulted in an overall methodological assessment of effectiveness in nine sections (see Appendix A).

## 3. Results

### 3.1. Description of the Studies Included

The literature search yielded 1589 published references. After reviewing the title, abstract, and full text of the records, a total of 12 studies met the full inclusion criteria and were included (see Table 1). All included studies were published between 2005 and 2021. Analysis by country showed that the largest scientific production in this field was developed in the USA (n = 10), followed by two European countries, namely the Czech Republic (n = 1) and Switzerland (n = 1).

Most of the included studies had a quantitative approach (n = 11) [60,61,62,63,64,65,66,67,68,69,70,71] and one had a qualitative approach [72]. Four studies were RCT (two had a cross-over design), one had a quasi-experimental design, four were before–after studies, one was a mixed-method study, one had a cross-sectional design, and one relied on qualitative data (Table 1).

In 11 studies, the intervention consisted of interaction between a trained or certified therapy dog and health care providers, whereas the study by Hediger et al. [67] involved a variety of animals. In two studies, AAI was conducted in a room separate from clinical care [60,61], whereas in the study by Yordy et al. [63], the dog had access to the ward and break room of the cardiovascular unit and to all areas of the nurses’ station in the medical clinic. In the Jensen et al. [65] study, health care workers were taught how best to work with dogs in their facility during a two-week partnership session. The dogs varied in size, age, and breed, but all were trained and/or certified to conduct therapy work.

Three studies measured the impact of AAI/AAT on burnout risk or its key dimensions (EE and PA) and compassion fatigue using standardized questionnaires (Copenhagen Burnout Inventory—CBI, Maslach Burnout Inventory—MBI) [62,63,64,65]. Ginex et al. [62] examined the impact of animal-assisted therapy on the psychological well-being of staff in a surgical oncology ward. Staff scores on compassion fatigue and burnout did not change significantly after the intervention, but a nonsignificant decrease in the staff burnout levels was observed. Another study by Clark et al. [63] of 24 nurses in the Department of General Internal Medicine who interacted with dogs during their work showed that therapy dog visits appeared to reduce compassion fatigue more than the control group without therapy dog visits. The positive effect was more pronounced when therapy dog visits were more frequent (twice a week for four weeks versus once in four weeks). In addition, therapy dog visits helped nurses to cope with burnout and feelings of being drained and overwhelmed by work.

In the mixed-method study by Etingen et al. [64], a small group of American multidisciplinary nurses and physicians showed better real-time mood scores (*p* = 0.0001) and lower patient-related burnout scores (*p* = 0.002) after participating in AAI programs. In the Jensen et al. [65] study, pediatric nurses who worked in a children’s hospital with a pet dog felt greater personal fulfillment at work (key dimension of burnout) and reported more positive descriptions of work, greater work-related enthusiasm, and less work-related depression than the control group who worked without a facility dog.

Other studies examined the effects of AAI/ATT on various work-related aspects and nurses’ well-being and job satisfaction [65,66,67,68,69,70,71].

Health care workers in a cardiovascular intensive care unit (CVSU) who participated in a five-week AAI program reported reduced stress levels and improved psychological well-being [66]. Health care workers at a Swiss rehabilitation clinic who worked with a variety of animals also showed increased job satisfaction and enrichment. In addition, the animals provided a tool for coping with daily stressful events on the ward [67]. Working with a pet dog was associated with a lower intention to leave the job and a greater intention to remain in the current position. In the Jensen et al. [65] study, working with a facility dog was also significantly associated with better self-reported mental health in the form of more positive affect related to recent experiences, less depression, lower negative affect, and better perceptions of mental health among personnel. Another study conducted with staff in two inpatient psychiatric units found that AAI can have a significant positive impact on mood change and alleviate “anxiety”, “anger”, “fatigue”, and “tension” [68]. Among staff at a burn center, the implementation of a dog therapy program resulted in improved mood following dog visits and increased job satisfaction [69]. Barker et al. [70] found that interaction with therapy animals may have a “stress-buffering effect” by reducing healthcare workers’ stress responses, as measured by physiological parameters, such as serum and salivary cortisol. Abrahamson et al. [71] found positive effects of animal-assisted therapy on hospital staff, who reported lower stress levels and improved relationships with patients.

### 3.2. Study Quality Evaluation

The results (see Appendix A) show that, in all of the selected papers, cause and effect were clear, none of them used participants who were included in similar comparisons, and none of the comparisons included participants who received similar treatment/care. Four studies had a control group, and eight had pre- and post-measurement. All studies included in the systematic review answered positively to the questions on follow-up, outcome measurement, and reliability. In one case, the question on statistics was answered ‘not applicable’.

## 4. Discussion

This review aimed to examine the psychological impact of AAI on the psychological well-being of HCWs. Animal-assisted interventions are often used as a complementary therapeutic intervention in clinical settings to promote the healing and rehabilitation of patients with acute or chronic illnesses [50,51]. The studies included in our review showed that lower levels of cortisol, which is a biological indicator of work-related stress in an occupational context, as well as lower levels of BOS and better levels of perceived psychological well-being and real-time mood, may be manifestations of lower levels of work-related stress. However, other benefits of AAI in healthcare workers relate to the areas of relationships and interpersonal communication, such that their empathy towards colleagues and patients may increase. Animals may, therefore, lead HCWs to engage effectively with their patients [72]. Stern and Chur-Hansen [73] found that AAI has long-term, positive effects on healthcare workers’ mobility, social contacts, and communication. As Chandler et al. [74] pointed out, AAI could support patients’ motivation and participation in counseling, and improve their sense of safety through a positive and “reassuring” effect, which could lead to improved patients’ adherence to treatment. In addition, AAI support programs may be positively impactful on workforce morale, which may in turn aid in improving the quality of care and service they provide to patients and ultimately patients’ experience with care. Brown et al. [68] found that AAI affected the mood and perceptions of feelings such as happiness, relaxation, and calm, while Abrahamson et al. [70] found that AAI increased patients’ promotion of social interactions and perceptions of comfort and companionship. The results showed that the dimensions of perceived personal accomplishment, work-related burnout, work perception, and mental health were higher among participants in AAI than in the control group. At the same time, HCWs who participated in AAI expressed less depression. However, AAI had no effect on emotional exhaustion or anxiety. Etingen et al. [64] also reiterated the findings from this literature and showed that AAI had a significant impact on mood and lowered the levels of patient-related burnout. Similar results were obtained by Clark et al. [63]; AAI can help combat exhaustion, straining, and frustration.

AAI is feasible and accepted by HCWs as they improve HCWs’ job satisfaction and clinic atmosphere (see also [75]). Similar findings were reported by Hediger et al. [67], who examined the concerns of HCWs prior to the implementation of AAI. These concerns were related to hygiene and injuries. These concerns were also found in other organizational areas. Workers might complain of negative feelings and perceive the presence of a pet as interfering with their well-being. In some cases, pets can trigger allergic reactions in susceptible individuals [76] or cause injuries [77], for example, a person might trip over the pet. When multiple pets live in a workplace, they may fight and cause harm [78]. This could pose legal problems for a company and damage the reputation of an organization [79]. In Barker et al.’s study [80], one fifth of employees without a dog reported that their presence in the workplace affected their productivity. According to some researchers, pets can be a distraction both at work and when working from home, as pets need to be groomed, fed, etc. during work hours [73]. However, Hediger et al. [67] reported that HCWs expressed potential problems before, rather than after, the introduction of AAI.

This systematic review had some limitations. The first limitation of this review is that we did not include studies on the effect of AAI in other contexts, such as military personnel [81]. In addition, most of the studies included in our review were cross-sectional. Subsequent longitudinal studies could better explain the mechanisms leading to human–animal interactions in the workplace and the consequences for human and animal well-being. However, our review improves knowledge about the positive and negative aspects of using AAI as a prevention strategy in HCWs. This knowledge could be considered by organizations adopting pet-friendly policies as well as health care workers looking to adopt a pet. Workplace health promotion programs could indeed use AAI programs to improve the mental well-being of HCWs and other categories of workers in combination with occupational health surveillance to prevent WRS.

## 5. Conclusions

The implementation of an AAI program in a busy clinic is feasible and the program is acceptable to HCWs due to the immense psychological benefits it offers. However, HCWs disliked the experimental design that required them to leave their work area at a prescribed time. We believe that an ideal design to reduce provider stress would be better described as a “dog on demand”. In future research, we are planning a paradigm that allows providers to interact with a therapy dog in or near their workspace whenever they wish, at least during part of their shift, and for the same dog to be available to patients experiencing stress. A future literature review could consider AAI in military personnel, particularly as a tertiary prevention strategy to increase the chance of intervention to alleviate PTSD symptoms. Qualitative studies should also be conducted to explore the potential negative effects of AAI in workplace settings.

## Figures and Tables

**Table 1:** Characteristics of the studies included in the review (n = 12).

Reference	Study Design	Country	Population	Study Conditions	AAI Describtion	Study Outcome (Measurement Tool)	Results in AAT/AAI Group (Data Collection Pre and Post-Intervention)
Machová et al., 2019 [60]	RCT with cross-over design	Czech Republic	Nurses (n = 22) PRM (n = 13) IML (n = 9) (women, mean age 30 y)	1- working day without break (A) 2- working day with break of choice (B) 3- working day with AAT (C)	Active- 20 min visit during work shift in a room separate from clinical care. HCWs may feed, pet or play with the dog, or sit/lie next to the dog	HCWs stress experience (SalC measurement)	↓ SalC level in condition C (*p* = 0.02) ↓ SalC levels in IML nurses (*p* = 0.02)
Kline et al., 2020 [61]	RCT	Indiana, USA	ED physicians and nurses (n = 122) (mean age 32 y, 58 women 53 men)	1- coloring activities (n = 40) 2- AAI (n = 43) 3- no activities (n = 39)	Active- 5 min visit halfway through work shift in a room separate from clinical care. HCWs may feed, pet, or play with the dog	HCWs stress experience—self-reported anxiety (VAS, mPSS-10) and objective SalC measurement (t1 beginning of shift, t2 30′ post-intervention, t3 end of shift)	VAS score in HCWs that interacted with dog ↓ from t1 to t3 (*p* = 0.015). ↓ SalC level in HCWs that interacted with dog “AAI” group vs. controls (*p* < 0.05)
Ginex, 2018 [62]	Before–after study	USA	HCWs in a surgical oncology unit (n = 41)		Animal-facilitated therapy—six weeks duration—every day Tuesday–Friday both direct and indirect interaction with animal	Job satisfaction, compassion fatigue and secondary trauma, burnout, and compassion satisfaction. (Professional Quality of Life Scale (ProQOL-5))	Increase in compassion Compassion satisfaction (*p* = 0.265) Burnout at baseline was 19.8 and at follow-up was 18.6 (*p* = 0.063) Qualitative findings revealed that staff viewed the AFT intervention positively
Clark, 2018 [63]	RCT	Minnesota, USA	Nurses in department of General Internal Medicine (n = 24)	Therapy dog: 15 min session with certified therapy dog on different schedule (treatment A: 2 visits/week, treatment B: 1 visit/week, treatment C: 1 visit every 2 weeks, treatment D: 1 visit every 4 weeks) vs. Treatment E no therapy dog visit	Petting therapy dog and conversing with the dog handler	Burnout (MBI-HSS), job satisfaction (NWSQ, NWI), anxiety (VAS).	Treatment B reduced depression (*p* = 0.02) and improved emotional well-being (*p* = 0.04). Treatment A improved happiness (*p* = 0.01). Treatment A reduced burnout (*p* = 0.04)
Etingen et al., 2020 [64]	Mixed method study with explanatory sequential approach Data collection pre-during and post-intervention	USA	Medical staff (n = 22 filled out pre-implementation-survey, women 17 65% < 49 y; n = 16 filled out post-implementation survey, women 15 50% < 49 y)		Active- 1 h session in a dedicated conference room performed in alternating weeks for 3 months. HCWs may observe, pet the dog or chat with the dog handler	Personal, work-related, and patient-related burnout (CBI), real-time mood (VAS)	Significant ↑mood in HCWs pre to post-intervention (*p* = 0.0001). Significant ↓ in patient-related burnout pre to post-intervention program (*p* = 0.002); NS ↓ in work-related burnout (*p* = 0.38) and personal burnout (*p* = 0.40) pre to post-intervention program
Jensen et al., 2021 [65]	Cross-sectional study	Georgia, USA	Pediatric healthcare professionals (n = 130) (mean age 37 y, women 92%)	1- working with a facility dog group (n = 65) 2- standard routinary activity group (n = 65)	Active- working with a facility dog during medical routine	Work-related burnout (MBI), job perceptions (JRDES, JDI, JIG, WSS, ATS, TIS), and mental well-being (PROMIS; SPANE)	Working with a facility dog is associated with ↑ PA (*p* < 0.001), ↑ job-related enthusiasm and ↓ job-related depression (*p* = 0.005), ↑ perception about the job overall (*p* = 0.004), ↓ willingness to retire early (*p* = 0.006) or quit the job (*p =* 0.002), ↓ depression (*p =* 0.025), ↑ overall mental health (*p* = 0.011), ↑ positive affect (*p* < 0.001), ↓ bìnegative affect (*p =* 0.031) than controls
Yordy et al., 2020 [66]	Before–after study	USA	Staff members of a cardiovascular unit (n = 79) 27 completed pre-intervention survey, 30 completed post-intervention survey		Active- 1 h visit during day or night shift for a 5-week period. Dog remained leashed at all times during the scheduled visits and was allowed access to the unit, the breakroom of the CVSU, and all parts of the nurses’ station at the medical clinic. The handler did not interact spontaneously with staff. Staff could freely interact with the dog	HCWs stress experience and mental well-being (BAATA Test)	↓ stress and ↑ overall wellbeing
Hediger et al., 2017 [67]	Before–after study	Switzerland	Staff members of a clinic of neurorehabilitation and paraplegiology (n = 165), 103 completed pre-intervention survey and 165 completed a post-intervention survey	AAT for patients with various health problems in rehabilitation programs	1-year period, various animals (horses, donkeys, goats, sheep, mini-pigs, chickens, rabbits, guinea-pigs, cats, birds, and dogs)	HCWs attitudes toward AAT HCWs stress experience, job satisfaction (BAMI–TGT)	↑ job satisfaction and enrichment, ↓ work stress
Brown et al., 2020 [68]	Before–after study	USA	Staff psychiatric department (n = 28) (ADU adult inpatient unit-n = 20; ALU adolescent inpatient unit-n = 8)		Session with a therapy dog	Mood change (VAMS, visual analog mood scale)	ADU staff A significant lower negative score for the moods “Afraid,” (b value = 0.034) “Angry” (b value = 0.038) “Tired” (b value = 0.034) and “Tense” (b value = 0.009) ALU staff no change in mood
Pruskowski et al., 2020 [69]	Quasi-experimental study	USA	Staff employed in Army Burn Center (intensive care unit, ward, and outpatient) (n = 23)		Session with a therapy dog	Job satisfaction, mood change (ad hoc questionnaire)	↑ staff mood and ↓staff stress level
Barker et al., 2005 [70]	RCT with cross-over design	USA	Nurses, physician, occupational therapists in medical inpatient services (n = 20) (mean age 38.6y women 95%)	1–20 min of quiet rest 2–5 min interactive activity with therapy dogs 3–20 min interactive activity with therapy dogs	No details	HCWs stress experience (serum and Sal cortisol measurement at 0, 5′, 15′, 30′, 45′, 60′ post-intervention)	Serum cortisol ↓ in all three conditions (*p* < 0.05), Sal cortisol ↓ in all three conditions (*p* = 0.004), Sal cortisol ↓ in all three conditions 5 min interaction with the dog was associated with cortisol reduction equivalent to a 20 min intervention or 20 min of quiet rest
Abrahamson et al., 2016 [71]	Qualitative study	USA	Hospital staff and volunteers employed in a medical and surgical community hospital	Four staff nurses three hospital staff members (welcome desk receptionist, unit clerk, program manager), and two hospital volunteers.	Session with dogs, 1/2 a week, duration: 15 min per visit during the workday	Stress, social interactions, and interaction with patients	↓staff stress level, ↑social interactions with patients

Notes: ↓ Decreased; ↑ Increased; ATS, anticipated turnover scale; BAATA test, Brisbane AAT acceptability test; BAMI-TGT, Basler Mitarbeiterfragebogen Tiergestützte Therapie; CBI, Copenhagen burnout inventory; ED emergency department; ILC, department of internal medicine and long-term care; JRDES, job-related depression–enthusiasm scale; JDI, job descriptive index; JIG, job in general; MBI, Maslach burnout inventory; PA, personal accomplishment; PROMIS, patient-related outcome information system; PRM, department of rehabilitation and physical medicine; SalC, salivary cortisol, SPANE, scale of positive and negative experience; TIS-6, turnover intention scale; and WSS, workplace social support.

## Data Availability

Not applicable.

## Appendix A

Study quality evaluation (Appendix A).

**Table A1:** Check-list to assess the quality of the study included in the systematic review.

References	Q1	Q2	Q3	Q4	Q5	Q6	Q7	Q8	Q9
Machová et al., 2019 [60]	Yes	No	No	No	Yes	Yes	Yes	Yes	Yes
Kline et al., 2020 [61]	Yes	No	No	Yes	Yes	Yes	Yes	Yes	Yes
Etingen et al., 2020 [63]	Yes	No	No	No	Yes	Yes	Yes	Yes	Yes
Jensen et al., 2021 [65]	Yes	No	No	Yes	No	Yes	Yes	Yes	Yes
Yordy et al., 2020 [66]	Yes	No	No	No	Yes	Yes	Yes	Yes	Yes
Hediger et al., 2019 [67]	Yes	No	No	No	Yes	Yes	Yes	Yes	Yes
Brown et al., 2020 [68]	Yes	No	No	No	Yes	Yes	Yes	Yes	Yes
Pruskowski 2020 [69]	Yes	No	No	No	No	Yes	Yes	Yes	Yes
Abrahamson et al., 2016 [71]	Yes	No	No	No	N/A	Yes	Yes	Yes	N/A
Barker, 2005 [70]	Yes	No	No	No	Yes	Yes	Yes	Yes	Yes
Clark, 2018 [64]	Yes	No	No	Yes	Yes	Yes	Yes	Yes	Yes
Ginex, 2018 [62]	Yes	No	No	Yes	No	Yes	Yes	Yes	Yes

Note. Q1: Is it clear in the study what is the ‘cause’ and what is the ‘effect’ (i.e., there is no confusion about which variable comes first)?; Q2: Were the participants included in any comparisons similar?; Q3: Were the participants included in any comparisons receiving similar treatment/care, other than the exposure or intervention of interest?; Q4: Was there a control group?; Q5: Were there multiple measurements of the outcome both pre and post the intervention/exposure?; Q6: Was follow up complete and if not, were differences between groups in terms of their follow up adequately described and analyzed?; Q7: Were the outcomes of participants included in any comparisons measured in the same way?; Q8: Were outcomes measured in a reliable way?; Q9: Was appropriate statistical analysis used?. Possible answers: Yes, No, Unclear (U) or Not/Applicable (N/A).
